# Navigating the complexities of out-of-state abortion training in the United States Post-*Dobbs*

**DOI:** 10.1016/j.xagr.2026.100676

**Published:** 2026-07-18

**Authors:** Kristyn Brandi, Emily Claymore, Kristin Simonson, Jema Turk, Jody Steinauer

**Affiliations:** Ryan Residency Training Program, University of California, San Francisco (UCSF), San Francisco, CA (Brandi, Claymore, Simonson, Turk, Steinauer).

**Keywords:** abortion, Dobbs, medical education, pregnancy options counseling, training

## Abstract

**BACKGROUND:**

In the United States**,** the *Dobbs v. Jackson Women's Health Organization (Dobbs)* decision has implications not just for patient care, but for abortion training. Consequently, training programs in states with abortion bans are developing out-of-state training rotations by partnering with programs in abortion-accessible states. It is unclear what barriers exist for trainees, sending and receiving site faculty as they develop and manage out-of-state training rotations.

**OBJECTIVE:**

This study aims to understand the perspectives of trainees and faculty as they navigate the post-*Dobbs* landscape and develop out-of-state training relationships.

**STUDY DESIGN:**

This qualitative study interviewed trainees, faculty from states with abortion bans considering sending trainees for abortion training ("Sending Sites") and faculty from states supportive of abortion that are receiving trainees (“Receiving Sites”). Interviews were conducted over Zoom, transcribed, and interview transcripts were analyzed utilizing Nvivo software for thematic qualitative analysis.

**RESULTS:**

We interviewed 9 trainees, 8 sending sites faculty and 5 receiving sites faculty across the country. Faculty in states with abortion bans noted many barriers to establishing an out-of-state rotation including funding and legal support. Receiving sites were worried about integrating outside trainees and making sure they got enough experience without negatively impacting their home trainees’ training. Regardless of rotation length and level of participation, all trainees reported positive experiences with out-of-state training.

**CONCLUSION:**

Despite barriers to developing out-of-state training rotations, trainees value the opportunity to obtain abortion training. Efforts should be made to improve facilitation of out-of-state training opportunities in the post-*Dobbs* medical landscape.

**AJOG Global Reports at a Glance**

**Why was the study conducted?**

The goal of this study was to better understand the experiences of trainees and faculty in states with abortion restrictions and limited abortion training including barriers and facilitators to accessing training out of state.

**What are the key findings?**

We learned that many barriers exist to creating out-of-state training opportunities but regardless of the level of exposure, trainees that get out-of-state training opportunities report it to be a valuable experience.

**What does this study add to what is known?**

This is the first study assessing the barriers and firsthand experiences of trainees and faculty in the post-Dobbs abortion training landscape.

## Introduction

The World Health Organization considers abortion care an essential healthcare service,^1^ and FIGO, the international organization governing obstetrics and gynecology (OBGYN), as well as the US-based organization, ACOG, state that all obstetrician-gynecologists must have the skills to do an abortion in the setting of an emergency.^2^^,^^3^ In the United States, the Accreditation Council for Graduate Medical Education (ACGME) requires that abortion training be a part of OBGYN residency training.^4^ After the ACGME requirement was enacted, abortion training as a required experience increased from 12% of OBGYN programs in 1992 to 72% in 2020.^5^ While not required, abortion is essential training in other medical specialties such as family medicine and disciplines such as advanced-practice nursing, and this training is often not available. The 2022 US Supreme Court’s *Dobbs* decision, which overturned the constitutional right to abortion, drastically changed abortion training.^6^ Thirteen states have since banned abortion at all gestations with varying, limited exceptions, comparable to the 21 countries with such abortion bans.^7^^,^^8^ These states house 54 OBGYN training programs and over 1200 trainees.^9^ making it challenging for these programs to meet ACGME requirements and prepare their trainees for future practice.

The ACGME continues to require training and states, “if a program is in a jurisdiction where resident access to this clinical experience is unlawful, the program must provide access to this clinical experience in a different jurisdiction where it is lawful.”^4^ Since *Dobbs*, some programs in states with bans have initiated out-of-state training partnerships with programs in unrestricted states. Little data exist on post-*Dobbs* out-of-state training. One study reported that the Ryan Program, a national program that supports OBGYN abortion training, devoted an average of 14 hours of staff time facilitating each out-of-state partnership, an estimate not including residency program time.^10^ Noted logistical challenges included “negotiating contracts and agreements between institutions, coordinating rotation schedules with host institutions, supporting individual learners' applications, and making travel plans.”^10^

We sought to explore the challenges in training learners and establishing partnerships through interviewing faculty and trainees in programs in states with bans on abortion and those without bans. These data will inform resource development to facilitate training opportunities, relevant for US programs and in countries with abortion bans throughout the world.

## Materials and methods

### Research design and recruitment

This study is part of a larger landscape analysis conducted in 2023 to 2024 to understand US abortion training, for which the research team interviewed a convenience sample of subjects involved in abortion training in academic or community clinical settings. The full project included interviews with faculty that were sending and receiving trainees from states with abortion bans, the trainees themselves, abortion clinics that were engaged in clinical training and representatives from larger organizations that were involved in medical education that included abortion. This analysis focused on interviews of faculty in training programs in states with abortion bans who were considering or sending trainees out of state (referred to as "Sending Sites Faculty, or SSF"), faculty in programs with fewer restrictions who were considering or receiving out-of-state learners ("Receiving Site Faculty, or RSF") and trainees in various healthcare fields with access to local abortion training or traveled for training (referred to as "Trainees"). Participants were over 18 years and spoke English. Participants were recruited via direct emails to faculty and trainees that were known by our team to be participating in out-of-state training opportunities We also sent general solicitation emails on listservs frequented by abortion educators, including the Ryan Program listserv. Snowball sampling was also used when participants had recommendations for potential participants. Of note, we conducted this study from May 2023-May 2024 when out-of-state partnerships were just starting to be developed.

Semi-structured interviews included several domains: status of abortion care and training, post-*Dobbs* challenges and efforts to improve/sustain abortion training. In sites where abortion was illegal, sites and trainees were asked about the status of, barriers to, and experiences of out-of-state training. For participants based in states where abortion was legal, we asked about their decision-making process around accepting out-of-state trainees and challenges with hosting.

### Data collection

Participants gave informed consent prior to beginning the interview. Interviews were conducted remotely through Zoom® by trained researchers (KB, EC, KS) using a semi-structured interview guide. Interviews were recorded via a voice recorder with Zoom recordings as backup. After each interview, data were de-identified and stored digitally in a password-protected online folder. Participants received a $50 gift card as compensation. De-identified audio recordings were sent to a third-party service for transcription. This study was deemed IRB exempt by the University of California San Francisco (UCSF) IRB.

### Data analysis

Interview transcripts were analyzed utilizing Nvivo. An initial codebook was developed utilizing inductive coding. Three researchers (KB, KS, EC) independently coded each transcript and discussed coding patterns until consensus was achieved regarding final codebook. The remaining transcripts were coded by a single researcher (KB). Interviews and analyses were continued until theoretical sufficiency was achieved within each group of participants. Researchers then synthesized the codes into themes.

## Results

In this cohort, we interviewed 22 people including faculty (OBGYN & family medicine) and trainees (OBGYN & family medicine residents, and nursing learners). See Table 1 for participant details. Notably, we had one “sending” and one “receiving” site that could not send/receive trainees. Our participants were mostly white (68.2%), non-Latinx (100%), and women (100%), and 36% were from the South and 9% from the Midwest. Over half of trainees interviewed had traveled for training.

**Table 1:** Demographics of faculty members in OBGYN training programs in abortion restrictive and abortion permissive states and health professions trainees in restrictive states

Characteristics	Total (n, proportion) (N=22)
Type of participant	
Trainees	9 (41%)
Sending site faculty	8 (36.3%)
Receiving site faculty	5 (22.7%)
Gender	
Women	22 (100%)
Race	
Asian	4 (18%)
Black	2 (9%)
White	15 (68%)
Mixed race	1 (5%)
Ethnicity	
Hispanic/Latina	0
Geographic region	
Midwest (WI)	2
Northeast (MA, NY, ML)	6
South (SC, GA, AL, DC, TX, OK)	8
West (CA, WA, UT)	6

We identified three themes: (1) uncertainty and pressure created by the *Dobbs* decision, (2) navigation of complex logistics, and (3) tension between rotation goals of exposure vs competency.

Theme 1 *Uncertainty and Pressure Created by the Dobbs Decision*

After the *Dobbs* decision, trainees and faculty in states with bans reported uncertainty about the future of training. OBGYN participants in both policy settings described challenges meeting the ACGME mandate; one faculty said, “The ACGME still requires [abortion training]. There are going to be lots of programs that can't meet that requirement, and we don’t know what’s going to happen.” (RSF1).

Many trainees noted that they were initially concerned about what training would look like when the *Dobbs* decision came down. Some had chosen their field or program based on the opportunity for abortion training. One OBGYN resident described the conflict as they decided where to apply for residency training:

“I was actually couples-matching with my husband… our main goal was to end up together. However, at the same time… I knew I did not want to practice in a state where I would not be able to provide this essential piece of health care. I didn't apply to any programs [in states] where laws were getting more and more stringent. (T2, training in a less restrictive state)

Faculty in programs where abortion became banned expressed that *Dobbs* created turmoil, made recruiting trainees difficult, and also inspired a training commitment. One said, “there was a shock after the *Dobbs* decision and scrambling” (Sending Site 3). Others had started planning even before *Dobbs*. In some circumstances, programs that had already struggled to create training opportunities found that the *Dobbs* decision gave them more momentum to expand training (see Table 2 for quote) or solidify education. One faculty said: “We were very clear that we were not changing our didactics; we were not changing our simulation… making it clear that our ban did not state that we needed to stop doing any of that.” (SSF8)

**Table 2:** Example quotes from each theme

Theme	Sample quote
Theme 1: uncertainty and pressure created by the Dobbs decision	“So, in anticipation of [*Dobbs*], we were prepared. We were talking to our GME office about creating an away rotation [pre-*Dobbs*]. And then, with the *Dobbs* decision, that was all accelerated. I was very happy with the ACGME statement that residents really must be trained in abortion. Because that was the leverage that I needed to even get in meetings with my GME to have conversations about this.” (Sending Site Faculty 7)
Theme 2: Navigation of complex logistics	“Well [the sending institution’s administration] were like, ‘New York has really expensive malpractice insurance: New York, California, Illinois. Maybe you should find different states.’ And [we] were like, ‘Those are the states that can do this [training]’.” (Receiving Site Faculty 3).
Theme 3: Tension between rotation goals of exposure vs competency	“I think it helps that their Ryan program director had sent me [their goals for the residents]. And she's like, ‘Obviously, they're not going to be competent in D&E, being there for two weeks, sharing procedures with your residents. That's not the goal…the goal is for them to be exposed to this stuff, to understand how it works, to do counseling, to do medication abortion, to feel like they get it, so that they know what it means. They can counsel patients about what's going to happen when they go to other places. And they know enough to know that they want more training after residency.’ It was kind of just set up like, whatever you can do is great…. I think that, knowing that we're not solely responsible for training people to be able to provide care in just this little two-week snippet it makes it lower stakes. I think that really took a lot of the pressure off.” (Receiving Site Faculty 3)
Theme 4: Managing the needs of trainees	“[Working with the home program’s residents] was great. It was really interesting to get a sense of how their day-to-day and their practice differs from our practice, and I think they learned a lot about… how things are in [a restricted state].” (Trainee 3)

Receiving sites also used the *Dobbs* decision as an opportunity to expand training. One program described that the *Dobbs* decision “potentially accelerated or protected a trend [of expanding family planning services] that was already in progress” and considered taking an out-of-state trainee as a “good practice to think about how to incorporate multiple learners [for a potential Complex Family Planning fellowship]” (Receiving Site 3). Another site used *Dobbs* to look at their clinical case numbers and determined that “there’s plenty to go around,” deciding to take an outside trainee (RSF4).

Lastly, trainees and faculty in non-OBGYN fields became concerned about the limited options that would be available for training in the post-*Dobbs* landscape. One family medicine trainee described:

“I worry that there's going to be a lot less training for family med, and I'm worried that we’ll need [extensive] training and fellowship [to be hired in abortion care]… and we only have five fellowship locations…. It feels like that, and I know it's already limited for my friends in OBGYN, too.” (T5)

Theme 2 *Navigation of Complex Logistics*

Faculty in sites sending trainees described having faced many barriers to creating partnerships for out-of-state training. One said, “I think people are trying to find solutions… and some of us are hitting roadblocks” (SSF5). Many sending-site faculty struggled with navigating logistics in creating these complex arrangements and faced resistance from academic leaders and hospital lawyers. One sending site described the burden as "the limiting factors of getting our visiting residents up and running has often been logistics,” including “templates for letters of agreement. … understanding what the rules are in your state for temporary licensure, malpractice, budget templates” (SSF3). These challenges took significant time and resources to arrange and were often done by the sending-site faculty without support staff. Throughout the process, sending sites were frustrated when barriers arose because of misunderstandings of what was needed (Table 2).

Logistics included identifying a training site, developing training contracts, obtaining medical licenses, obtaining liability coverage and funding travel costs. Programs that had difficulty identifying a site did not know where to go other than reaching out to the Ryan Program. One program director described this as a snowball effect: “So it really just started with, ‘Okay, who do we know, and who can we reach out to that could potentially host residents?’ And so, we all just kind of emailed folks that we knew” (SSF6). Once a site was identified, many reported that it was difficult to find support within their GME office and legal department to craft a Program Letter of Agreement (PLA) and get financial support for malpractice. One program said “Our GME office was just like, really, really not cooperative. … none of that seemed like problematic on the [supportive state] side. It was just my GME office, just kind of being obstructionist towards getting the money ready, the liability insurance” (SSF7). Another program described the malpractice being the hardest step: “I think that figuring out the malpractice insurance, figuring out what paperwork everyone was going to require—I think those were the biggest, and it was mostly administrative delays, honestly” (SSF2). Another described getting the training license as the most onerous component: “The licensing was a huge barrier and continues to be a huge headache…we’ve had residents miss their scheduled block because the license didn’t come through in time” (SSF3).

When asked about how successful partnerships were developed, many reported that having institutional support was critical. Specifically, support from chairs, designated institutional officers (DIOs) and outside organizations helped both with logistics and financial support. One site said: “I am very grateful to the Ryan Program that they funded the first [traveling] residents….and then, fortunately, our chair’s been able to keep up that support. But I'm very concerned that if we did not have that…I don’t know that this would have happened” (SSF2).

Some trainees and training sites had barriers so onerous that they could not overcome them. One program reported being unable to send residents because they could not navigate the legal barriers: “I had met with [a site for training]. And they had availability for our residents and were willing... that was not the tricky part. The hard part is figuring out how to get our residents there with the current legal landscape of [our] state.” (SSF5)

The family medicine faculty member and the family medicine and nursing trainees experienced barriers due to prioritization of OBGYN residency training. The trainees described hurdles in accessing training, reporting an unspoken competition for abortion training opportunities and that most were going to OBGYN trainees. One family medicine resident described:

“It's hard to get hands-on training... I feel sometimes uncomfortable talking about that in an OB community... I definitely have been made to question my skills or rights to do certain procedures in a lot of OB-heavy environments, which I think is probably similar to how APCs feel, but it sort of feels like this family med is in this weird in-between… we get the “doctors taking all the training slots” [concern from APCs] but also it's hard for [family medicine] to [get training] too.” (T5)

One person described that it may get worse as fewer spots are available:

“There's also a lot more focus on training in our OBGYN colleagues. I think the family med folks and their space for abortion training is being a little bit threatened and compromised, and I worry that that will be even further compromised in the future.” (T7)

Theme 3 *Tension between Rotation Goals of Exposure vs Competency*

Both sending and receiving faculty grappled with rotational goals of "exposure vs competency" in specific skills given the realities of short, away rotations. Trainees learned a variety of clinical skills, including counseling, ultrasound, pre- and postoperative assessment, medication abortion management, and first- and sometimes second-trimester abortion procedures; depending on the site, in some of these skills they gained exposure and in some competency. Exposure was considered to include minimal hands-on training deemed inadequate to achieve competency in abortion procedures. Competency was interpreted as adequate clinical skills training for independent practice of procedural abortion after the rotation. One trainee felt that gaining competency meant “that I could safely do a procedure and manage complications” (T5). Another described competency as “ready to independently practice by yourself…and you are able to manage the complications associated with the procedure, that you also are able to adequately counsel your patients” (SSF 4).

Many faculty described wanting the trainees to obtain a meaningful experience but were unclear whether the goal should be exposure or gaining competency to provide care without additional training. Generally, sending sites wanted trainees to become competent in all abortion skills including in the second trimester. For example, one program described their “hope would be that everyone would be competent in at least early second-trimester D&Es” (Sending Site 5). Some receiving sites felt that they would not be able to meet the sending sites’ goals in a short time frame and within their constraints.

Some faculty described that even if they don’t achieve competency in all skills, trainees would gain valuable skills they could use and build on in the future. One receiving-site faculty member said: “I would love to have more volume... But because they're coming as third-year residents, they're able to make a lot out of the small volume that they do have. …With some, learning the instruments, having a dozen to 20 cases under their belt, they could certainly be able to do uncomplicated first-trimester abortion and miscarriage management.” (RS4). Some receiving-site faculty worried that this was not achievable, especially considering they had to balance cases for the incoming trainees with cases for their home residents. But trainees working with outside trainees reported that this interaction was valuable to them as well, particularly sharing their skills and hearing how outside trainees manage logistics in their home states.

Critically, both trainees and trainers reported that regardless of how many cases trainees did, simply training in an environment that supports abortion was a new and valuable experience. By not focusing on the numbers and creating rotations that valued the whole experience, receiving-site faculty felt better about taking on a trainee. One receiving faculty articulated the benefit of having realistic learning goals set by the sending site, which alleviated the pressure of developing incoming resident skills to competency (Table 2).

## Structured discussion / comment

### Principal findings

After the US Supreme Court’s *Dobbs* decision led to states banning abortion, trainees became concerned about how to access training and educators about how to ensure adequate training for their learners. One solution for residency program faculty was to establish out-of-state training partnerships. Participants described that arranging out-of-state training rotations is logistically challenging and programs are in desperate need of support to navigate barriers. Barriers include finding a partnership, getting support within their department, garnering legal support, and travel funding. While trainees and sending sites would like hands-on training, rotations that were exposure-only are valuable experiences.

### Results

US trainees in reproductive health care fields desire abortion training and prioritize this in their application process.^11^^,^^12^ However, even before the *Dobbs* decision, many OBGYN programs did not offer this training, let alone in other disciplines.^13^^,^^14^ Trainees have expressed concerns about whether their training will be adequate to provide care and have conflicting emotions about whether to train in and practice in states with restrictions.^15^

There are limited data about the impact of the *Dobbs* decision on abortion training. Some have described how bans have limited training^16^^,^^17^ and have described the process of developing an out-of-state rotation for abortion training.^18^ In a brief report, we previously documented the quantitative experiences of 60 residents that traveled from 13 programs for training and some of the challenges that the Ryan Program had in setting up these partnerships.^10^ Our qualitative study adds to the literature by exploring the challenges of establishing training partnerships in more depth, with our participants describing numerous logistical, legal, institutional, and financial barriers.

There are many countries in which abortion is banned with no or rare exceptions,^8^ and in these and other countries, training is challenging due to insufficient clinical exposure. Training in these settings rely on a variety of strategies including a focus on medication abortion,^19^ implementation of and procedural training in postabortion care (when people have had an incomplete abortion) which can be applied to abortion care,^20^^,^^21^ and international convenings.^22^ To our knowledge there are no published descriptions of countries in which their learners travel to another country for training.

### Clinical implications

Abortion is a safe, common, and critical component of reproductive health care, yet there are significant areas of the US without sufficient abortion care providers. Because all OBGYNS must be able to provide abortion care at a minimum in the setting of an emergency and because abortion training prepares clinicians to provide patient-centered and high-quality abortion and pregnancy loss care, the ACGME continues to require abortion training within OBGYN residency programs. Moreover, training equips clinicians to manage rarer pregnancy complications such as previable, preterm rupture of membranes, abnormal placentation, and pregnancy of unknown location. Even if training sites are unable to train visiting learners to a level of competency in all abortion skills, the learning is valuable. Studies before *Dobbs* of OBGYN residents that chose to partially participate in an abortion rotation, and often did very few or no abortion procedures, reported valuing the rotation and improved knowledge and skills acquisition^23^^,^^24^ in a variety of aspects of care. We encourage the ACGME to continue to mandate training for OBGYN residents, and we encourage that OBGYN organizations develop resources and strategies for programs to overcome the many barriers described in our data.

Our study demonstrates that programs are struggling to ensure this training, but some are unable to train residents to competence in all skills during the away rotation. The concerns about the training rotations only being able to offer exposure for some clinical skills suggests that partnerships should have clear learning objectives, and consider the away rotation as part of their overall competency-based medical education structure, mapped to the ACGME milestones in OBGYN. Sites in restrictive states can build on their residents’ abortion skills by training them using simulation and in areas of OBGYN that train in overlapping skills such as in pregnancy loss care.^25^ We encourage programs to expand pregnancy loss care, for example, through developing Early Pregnancy Assessment Clinics.^26^

It is important to emphasize that, to meet the needs of patients, training is critical for the larger healthcare workforce, and that these training struggles are not limited to OBGYN. Efforts should continue to implement training requirements in other medical specialties and healthcare disciplines that include OBGYN care, such as family medicine and graduate nursing programs such as nurse practitioner and nurse midwifery training, as well as in undergraduate medical and nursing programs. Health professional training organizations in nursing and medicine should address these complex barriers.

### Research implications

Future research is needed to better understand the impact state-level abortion restrictions have on abortion training, ability to recruit trainees to restricted states, and post-training geographic practice decisions. This should include the diverse healthcare workforce. Tools to help programs develop sustainable training opportunities should be developed and evaluated.

### Strengths and limitations

Strengths of this study include having both trainees and faculty at sending and receiving sites, allowing for experiences from different angles and understandings of training barriers. Qualitative data allows for a deeper exploration of training than quantitative; however, because most participants were successfully engaging in a partnership, our data may not reflect more of those facing barriers. Our participants were mostly white women with limited geographic diversity, thus not reflecting the diversity of training programs; similarly, our data were primarily limited to OBGYN participants and did not reflect many in the reproductive healthcare workforce. We also acknowledge that this study was performed early in the post-*Dobbs* period, and as more programs have developed partnerships since then, the obstacles may have changed.

### Conclusions

Abortion training may become even more challenging as the ripples from the *Dobbs* decision continue. Too many clinicians face logistical barriers to getting abortion training. While many programs seek partnerships with sites that can train to competence, any experience - even those supporting early skill development - provides valuable transferable skills. Programs in areas where abortion is legal, regardless of volume, should consider how they can integrate outside learners within their institutions to improve access to abortion training.

## CRediT authorship contribution statement

**Kristyn Brandi:** Writing – review & editing, Writing – original draft, Methodology, Formal analysis, Data curation, Conceptualization. **Emily Claymore:** Writing – review & editing, Methodology, Conceptualization. **Kristin Simonson:** Investigation, Data curation, Conceptualization. **Jema Turk:** Project administration, Funding acquisition, Conceptualization. **Jody Steinauer:** Writing – review & editing, Funding acquisition, Conceptualization.
